# Spontaneous rupture of the urinary bladder with acute hepatic and renal failure: a case report

**DOI:** 10.1016/j.ijscr.2025.111279

**Published:** 2025-04-08

**Authors:** MA. Sobhi, M. Tetou, MA. Harchaoui, L. Hamedoun, M. Alami, A. Ameur

**Affiliations:** Urology Département, Mohamed V Military Hospital, Morocco

**Keywords:** Spontaneous rupture urinary bladder, Urological emergency, SRUB, CT cystography, Surgical management

## Abstract

**Background:**

Spontaneous rupture of the urinary bladder (SRUB) is a rare but life-threatening emergency that often presents with nonspecific symptoms, leading to delayed diagnosis and severe complications such as peritonitis, sepsis, and multi-organ failure. In rare cases, SRUB may present with multiorgan dysfunction including acute liver and kidney failure, complicating management and recovery. It is commonly associated with underlying bladder dysfunction, including chronic urinary retention and bladder outlet obstruction.

**Case presentation:**

We report a 58-year-old male with a history of benign prostatic hyperplasia (BPH) who presented with acute abdominal pain, fever, jaundice, and hemodynamic instability. Laboratory tests revealed leukocytosis, renal impairment, metabolic acidosis, and hyperkalemia, as well as elevated bilirubin and liver enzyme levels. Abdominopelvic CT with cystography confirmed SRUB, showing contrast extravasation. The patient underwent an urgent exploratory laparotomy, which revealed two bladder tears that were successfully repaired. Postoperative care included broad-spectrum antibiotics, hemodialysis, and intensive monitoring, leading to a full recovery.

**Discussion:**

SRUB is often misdiagnosed due to its resemblance to gastrointestinal and renal pathologies. This case highlights the importance of a high index of suspicion in patients with risk factors such as bladder outlet obstruction. CT cystography is crucial for early diagnosis, while surgical repair remains the gold standard for treatment. Supportive care, including hemodialysis and infection control, is vital for optimizing outcomes.

**Conclusion:**

Early recognition and prompt surgical intervention are critical in managing SRUB. Clinicians should consider this rare condition in patients with acute abdomen and known bladder dysfunction to reduce morbidity and improve prognosis.

## Highlights

• Spontaneous bladder rupture (SRUB) is a rare but life-threatening urological emergency.
• Delayed diagnosis increases the risk of peritonitis, sepsis, and multi-organ failure.
• CT cystography is the gold standard for detecting bladder rupture and contrast leakage.
• Early surgical intervention with bladder repair ensures optimal recovery and outcomes.
• Prompt diagnosis and multidisciplinary management significantly reduce morbidity.

## 1 Introduction

Spontaneous rupture of the urinary bladder (SRUB) is a rare but critical urological emergency, defined by the abrupt and non-traumatic breach of the bladder wall. It occurs in approximately 1 in 126,000 individuals and accounts for <1% of bladder injuries. Unlike trauma-induced cases, SRUB is commonly linked to conditions like chronic retention, bladder cancer, radiation, alcohol use, BPH, neurogenic bladder, or diverticula [1,2].

SRUB has a variable presentation, from mild abdominal pain to severe peritonitis, sepsis, or acute kidney injury, making misdiagnosis common. A recent review reported a 64% initial misdiagnosis rate, with mortality reaching 25–50% in delayed cases and 15% even with timely care [3]. Though uncommon, SRUB may cause acute renal and hepatic dysfunction, sometimes mimicking hepatorenal syndrome (HRS) without meeting its diagnostic criteria.

We report a rare case of SRUB with combined renal and liver failure, highlighting the need for early recognition in patients with abdominal symptoms and bladder rupture risk factors.

This work has been reported in line with the SCARE guidelines [4].

## 2 Case presentation

We present the case of a 58-year-old male patient with a history of BPH, managed with alpha-blockers with inadequate treatment compliance. His past medical history was otherwise unremarkable, with no known chronic liver disease or alcohol misuse, and no prior abdominal surgeries or trauma. The patient was admitted to the emergency room with a diffuse abdominal pain that had progressively worsened over the preceding 3 days. On admission, the patient presented in distress, displaying polypnea, a fever of 39 °C, tachycardia of 120 beats per minute, and extensive jaundice. The abdominal examination indicated widespread guarding, particularly in the right iliac fossa. No bladder distension was palpable on examination.

Laboratory investigations revealed a severe infectious illness, characterized by a white blood cell count of 33,100/mm^3^ and a C-reactive protein (CRP) level of 326 mg/l. Renal dysfunction was indicated by a creatinine level of 150 μmol/l and urea at 3.45 g/l, accompanied by significant electrolyte disturbances, including hyperkalemia (potassium 7 mmol/l) and metabolic acidosis (bicarbonate 8 mmol/l). Hepatic impairment was seen, characterized by cholestasis and cytolysis, with a total bilirubin of 180 μmol/l (normal < 20 μmol/l) and elevated liver enzymes (ALT 70 U/l, AST 66 U/l, ALP 425 U/l, GGT 275 U/l). Additionally, the electrocardiogram (ECG) demonstrated tall, symmetrical T waves in the anteroseptal leads, suggestive of hyperkalemia-related changes.

**Fig. 1:**
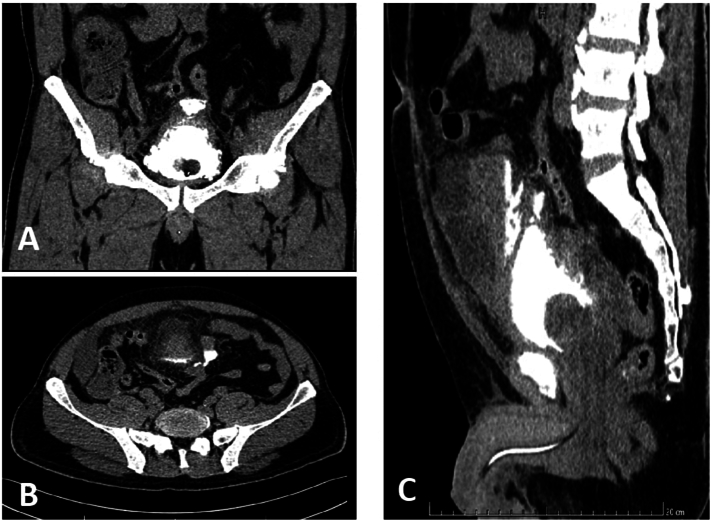
Abdominopelvic CT scan demonstrating contrast extravasation, a: Coronal view, B: Axial view, C sagittal view.

The patient received an abdominopelvic CT scan, including a CT cystogram, which demonstrated a bladder with several diverticula, an enlarged prostate (∼80 ml), and a localized rupture of the anterosuperior bladder wall, characterized by a 5 mm breach with contrast extravasation along with peritoneal effusion in the right iliac fossa and pelvis, and bilateral pyelocaliceal dilatation (Fig. 1). This imaging was obtained within three hours of presentation, confirming the diagnosis of generalized peritonitis secondary to urinary bladder rupture.

The patient received resuscitation measures, which included 2 l of intravenous crystalloid fluids, broad-spectrum antibiotic treatment (a third-generation cephalosporin, an aminoglycoside, and metronidazole), and emergency hemodialysis to mitigate life-threatening hyperkalemia and severe acidosis.

**Fig. 2:**
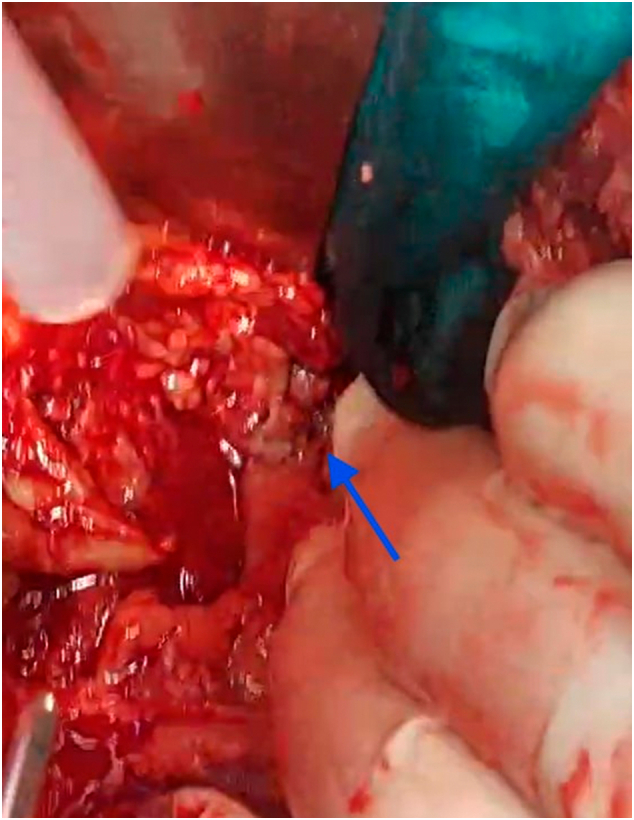
Intraoperative image showing rupture in the anterior bladder wall.

The patient was then sent to the operating room, approximately 6 h after his admission, for an exploratory laparotomy. A midline laparotomy traversing the umbilicus was conducted under general anesthesia. Approximately 2 l of peritoneal fluid were aspirated. Surgical examination revealed two tears in the bladder's anterior wall, which were fixed in two layers with 2/0 Vicryl sutures (Fig. 2). An intraoperative leak test confirmed bladder integrity, ensuring no further extravasation. A Foley catheter was left in place to divert urine and protect the repair. The abdominal cavity was meticulously irrigated, and drainage was secured with a Delbet drain positioned in the right paracolic gutter and a Redon drain in the pouch of Douglas.

The patient underwent a successful surgical recovery. Bowel function was restored on postoperative day 1. The Redon and Delbet drains were removed on day 3. On day 5, the WBC count fell to 16,000/mm^3^ and CRP reduced to 129 mg/l. Renal function showed considerable improvement, with creatinine levels at 31 μmol/l and urea at 0.8 g/l, Hepatic function also improved significantly as total bilirubin decreased to 43 μmol/l, and liver enzymes showed a progressive decrease, ALT dropped to 45 U/l, AST to 38 U/l, ALP to 110 U/l and GGT to 200 U/l. Bacteriological examination of the peritoneal fluid and urine cultures revealed *Escherichia coli*, which demonstrated susceptibility to the empirically administered antibiotic regimen.

The patient was discharged in stable condition on the sixth day with the Foley catheter in place and was prescribed a 14-day course of oral antibiotics (cefixime).

At two weeks, cystography confirmed bladder healing. The patient then underwent transurethral resection of the prostate (TURP) without complications. The catheter was removed on day two post-op, and ultrasound showed no post-void residual. At three-month follow-up, the patient was asymptomatic. Liver enzymes were normal (AST 14 U/l, ALT 15 U/l, GGT 20 U/l, ALP 35 U/l), creatinine was stable (25 μmol/l), and ultrasound showed complete bladder emptying.

## 3 Discussion

SRUB is a rare but serious condition that must be identified early and treated to avoid morbid outcomes. This case examines the management options, diagnostic techniques, and therapeutic implications based on current literature.

In this case, SRUB likely resulted from chronic bladder outlet obstruction (BOO) due to benign prostatic hyperplasia (BPH), worsened by poor treatment adherence. BOO leads to detrusor hypertrophy, urinary retention, and overdistension, key contributors to bladder wall weakening and rupture [1,5]. Bladder diverticula, often secondary to BOO, are structurally weak due to the absence of a muscular layer, making them potential rupture sites under pressure [6]. However, their exact role remains uncertain, as diverticulum location rarely aligns with rupture sites [7].

SRUB frequently presents with non-specific symptoms such as abdominal pain, sepsis, or renal impairment, complicating timely diagnosis. Bessa et al. described a female patient with ascites and peritonitis, ultimately diagnosed with SRUB during exploratory laparotomy [8]. Tariqi et al. reported SRUB secondary to bladder squamous cell carcinoma, manifesting as acute peritonitis [9]. Noë et al. (2023) emphasized the diagnostic challenge of SRUB in an atypical presentation when the patient initially presented with urinary retention, and the bladder rupture was only discovered intraoperatively. Interestingly, the rupture had been temporarily sealed by adjacent bowel loops, masking the typical signs and delaying diagnosis [10]. Additionally, Teressa et al. presented two cases of atraumatic bladder rupture due to chronic urinary retention and bladder wall weakness, reinforcing the role of underlying pathology and the importance of maintaining a high index of suspicion in similar clinical scenarios [6].

CT cystography is the gold standard for diagnosing bladder rupture, offering superior sensitivity over ultrasound or plain CT [11]. In this case, it confirmed the diagnosis early, facilitating prompt surgical intervention.

Surgical repair remains the standard for intraperitoneal bladder rupture, as endorsed by major urological guidelines. The American Urological Association (AUA) recommends prompt surgical intervention due to the risk of peritonitis and sepsis [12]. Similarly, the European Association of Urology (EAU) reaffirmed in its 2024 guidelines that immediate surgery is essential, especially in unstable patients or those with peritonitis [13]. Our patient underwent timely open surgical repair using a two-layer absorbable suture technique, commonly employed for better sealing in contaminated fields or fragile tissue. A Foley catheter was also left in place for continuous bladder decompression, reducing tension on the suture line and aiding healing. Both AUA and EAU guidelines recommend maintaining drainage for 10–14 days, with follow-up imaging (usually cystography) to confirm repair integrity before catheter removal [12,13].

A notable aspect of this case was significant jaundice with elevated liver enzymes. Sepsis-induced cholestasis, common in critical illness, is mediated by cytokines such as TNF-α, IL-1β, and IL-6, which disrupt bile transport, leading to intrahepatic cholestasis and raised conjugated bilirubin, ALP, and GGT [14]. Additionally, uroperitoneum from bladder rupture allows peritoneal reabsorption of urea, creatinine, and other solutes, a “reverse dialysis” effect, mimicking renal failure and promoting systemic inflammation and hepatic dysfunction [15]. In our case, the combined effects of sepsis and urinary ascites likely caused the observed hyperbilirubinemia and transaminasemia, as similarly described in recent SRUB reports [8,16,17].

To prevent recurrence, our patient was scheduled for definitive BPH management. Surgical relief of bladder outlet obstruction, such as TURP, is recommended to lower the risk of pressure-related complications like diverticula and rupture [12,13]. Early urological assessment and timely intervention are key to avoiding severe outcomes such as SRUB in high-risk patients.

## 4 Conclusion

This case highlights the significance of a multidisciplinary strategy in the management of SRUB, including prompt stabilization, imaging diagnosis, and timely surgical repair. Although uncommon, SRUB should be considered in the differential diagnosis of patients with acute abdominal symptoms, particularly those with a history of bladder dysfunction or outlet obstruction. Timely identification and appropriate intervention are crucial for an improved outcome, especially when complicated by concurrent organ failure.

## Consent

Written informed consent was obtained from the patient for publication of this case report and accompanying images. A copy of the written consent is available for review by the Editor-in-Chief of this journal on request.

## Source of funding

This study did not receive any specific grant from funding agencies in the public, commercial, or non-profit sectors.

## Declaration of competing interest

The authors declare no conflicts of interest regarding this publication.

## References

[1] Zhang Y., Yuan S., Alshayyah R.W., Liu W., Yu Y., Shen C. (2021). Spontaneous rupture of urinary bladder: two case reports and review of literature. Front. Surg..

[2] Ping L.S. (2021). Spontaneous rupture of bladder diverticulum: a case report of non-traumatic extraperitoneal urinary bladder rupture managed with surgical intervention. Int. Surg. J..

[3] Reddy D., Laher A.E., Lawrentschuk N., Adam A. (2023). Spontaneous (idiopathic) rupture of the urinary bladder: a systematic review of case series and reports. BJU Int..

[4] Sohrabi C., Mathew G., Maria N., Kerwan A., Franchi T., Agha R.A. (2023). The SCARE 2023 guideline: updating consensus surgical CAse REport (SCARE) guidelines. Int. J. Surg..

[5] Reddy D. (2023).

[6] Teressa SG, Parikh HR, Meng M, Mohseny A, Silletti J, Lehman D. Two Unique Cases of Atraumatic Bladder Rupture Consequent to Underlying Chronic Disease.

[7] Sung C.-W., Chang C.-C., Chen S.-Y., Tseng W.-P. (2018). Spontaneous rupture of urinary bladder diverticulum with pseudo-acute renal failure. Intern. Emerg. Med..

[8] Bessa I., Costa A., Ribeiro E., Leite L.M. (2025). Ascites secondary to spontaneous bladder rupture: a case report. Cureus.

[9] Tariqi R., El Abidi H., Boujida I., Darqaoui S., Boualaoui I., Ibrahimi A. (2025). Squamous cell carcinoma of the bladder as a cause of spontaneous bladder rupture: a case report. Afr. J. Urol..

[10] Noë C., Kouamé Y.E., Bolasade A.T., Michel T.L.S., Daouda Y.D., Vamoussa D. (2023). Spontaneous rupture of urinary bladder (SRUB): an exceptional presentation. Open J. Urol..

[11] Kunichika H., Takahama J., Taguchi H., Haga M., Shimoda E., Inoue M. (2023). The diagnostic challenge of non-traumatic bladder rupture: a pictorial essay. Jpn. J. Radiol..

[12] Morey A.F., Broghammer J.A., Hollowell C.M., McKibben M.J., Souter L. (2021). Urotrauma guideline 2020: AUA guideline. J. Urol..

[13] Serafetinidis E., Campos-Juanatey F., Hallscheidt P., Mahmud H., Mayer E., Schouten N. (2024). Summary paper of the updated 2023 European Association of Urology guidelines on urological trauma. Eur. Urol. Focus.

[14] Ghenu M.I., Dragoş D., Manea M.M., Ionescu D., Negreanu L. (2022). Pathophysiology of sepsis-induced cholestasis: a review. JGH Open.

[15] Matsumura M, Ando N, Kumabe A, Dhaliwal G. Pseudo-renal failure: bladder rupture with urinary ascites. Case Rep. Dermatol. 2015;2015:bcr2015212671. 10.1136/bcr-2015-212671 PMC4680254 26590189

[16] Shah Y.R., Dahiya D.S., Chitagi P., Rabinowitz L.G. (2023). Hyperbilirubinemia in a patient with Sepsis: a diagnostic challenge. ACG Case Rep. J..

[17] Putota G., Ziblim A.M., Wewoli B.A. (2025). Spontaneous rupture of the urinary bladder presenting as an acute abdomen: a rare case report. Clin. Case Reports.

